# Comparative Analysis of Antithrombotic Therapy Outcomes in Mild Traumatic Brain-Injury Patients: A Focus on Bleeding Risk and Hospital-Stay Duration

**DOI:** 10.3390/life14030308

**Published:** 2024-02-27

**Authors:** Antonio Desai, Dana Shiffer, Mauro Giordano, Alice Giotta Lucifero, Elena Generali, Francesco Reggiani, Marta Calatroni, Gabriele Savioli, Sabino Luzzi, Antonio Voza

**Affiliations:** 1 Department of Emergency Medicine, IRCCS Humanitas Research Hospital, 20089 Milan, Italy; dana.shiffer@humanitas.it (D.S.); elena.generali@humanitasresearch.it (E.G.); antonio.voza@humanitas.it (A.V.); 2 Department of Biomedical Sciences, Humanitas University, 20089 Milan, Italy; francesco.reggiani@humanitas.it (F.R.); marta.calatroni@humanitas.it (M.C.); 3 Department of Advanced Medical and Surgical Sciences, University of Campania “L. Vanvitelli”, 80138 Naples, Italy; mauro.giordano@unicampania.it; 4 Department of Brain and Behavioral Sciences, University of Pavia, 27100 Pavia, Italy; 5 Nephrology and Dialysis Unit, IRCCS Humanitas Research Hospital, 20089 Milan, Italy; 6 Emergency Department, IRCCS Policlinico San Matteo, 27100 Pavia, Italy; gabrielesavioli@gmail.com; 7 Department of Clinical-Surgical, Diagnostic, and Pediatric Sciences, University of Pavia, 27100 Pavia, Italy; sabino.luzzi@unipv.it; 8 Neurosurgery Unit, Fondazione IRCCS Policlinico San Matteo, 27100 Pavia, Italy

**Keywords:** anticoagulants, aspirin, elderly patients, intracranial bleeding, length of hospitalization, traumatic brain injury

## Abstract

Background: Traumatic brain injury (TBI) in the elderly is a noteworthy pathology due to the exponential increase in population age, and the effects of antiplatelet and anticoagulation on patients’ outcomes are still a matter of dispute. The aim of the present study was to evaluate the impact of various antithrombotic agents on patients with mild TBI, focusing on the risk of intracranial bleeding (ICH) and length of hospitalization (LOS). Methods: A retrospective analysis was conducted, including patients with a diagnosis of TBI admitted to the Emergency Department between 2021 and 2022. Patients were classified according to the concurrent antithrombotic therapy as aspirin (ASA), antiplatelets, direct oral anticoagulants (DOACs), and low-molecular-weight heparin (LMWH). The primary outcome was the ICH occurrence, while the secondary outcome was the LOS. The statistical analysis was performed via logistic regression models in R and STATA 13.1 software. Fisher’s exact test was used for the statistical significance. Results: 267 patients with mild TBI were included; 148 were not on antithrombotic agents, 43 were on aspirin, 33 on DOACs, 5 on LMWH, 22 on antiplatelets, and 16 on VKA. Out of the total, 9 patients experienced ICH, none of which were on DOACs, LMWH, or VKA, but 4—out of 65—were on antiplatelets, and 5—out of 148—were not on antithrombotic therapies. Patients not on antithrombotic therapy had the shortest LOS at 0.46 days, while those on VKA had the longest LOS at 1.19 days; similar trends were observed for patients on DOAC and LMWH. Conclusions: The results reveal that TBI patients on anticoagulants/antiplatelets had longer hospital stays compared with those on aspirin alone. Notably, VKA was the strongest predictor for an extended LOS. Regarding ICH, patients taking only aspirin were twice as likely to experience bleeding compared with those on anticoagulants/antiplatelets. However, to achieve statistically significant evidence, further research with a larger cohort of patients is needed.

## 1. Introduction

With the aging of the global population, an increase in the proportion of elderly patients presenting with traumatic brain injury (TBI), defined as a disturbance in the functioning of the brain or evidence of brain pathology that results from an external physical force, is expected [1,2,3]. Indeed, in recent years, instances of neurotrauma in the elderly have been increasing [4,5]. Given this aging population, one can expect to see certain trends, such as increased numbers of TBI due to falls in the elderly, and among TBI patients, increased use of antiplatelet and anticoagulation medications [6,7,8]. The age of afflicted patients has increased in the past decade, and the outcome for elderly patients is often worse, in part because of the widespread use of anticoagulants and thrombocyte inhibitors [9]. Consequently, understanding the effects of various antithrombotic agents on TBI outcomes has become critically important.

There are mixed results in the literature regarding the safety of direct oral anticoagulants (DOACs) compared to traditional agents like vitamin K antagonists (VKA) and antiplatelet drugs in TBI scenarios. While studies have shown that VKA patients have a 30–70% increased risk of intracranial hemorrhage (ICH) compared with those on DOACs [10,11], the literature on TBI outcomes is more conflicting [12,13,14,15]. Moreover, most of these studies were conducted before the FDA approval of DOAC reversal agents, such as adnexa alpha, which complicates the assessment of current TBI management strategies.

Previous research indicates that TBI patients treated with VKA experience higher rates of mortality and neurosurgical interventions [16,17,18]. For example, some studies demonstrated significantly higher mortality in VKA groups compared with non-VKA groups among older patients who sustained head injuries from falls [16,17]. Similarly, increased mortality and neurosurgical interventions were observed in patients on VKA [18]. However, other studies have shown conflicting results, suggesting the need for a nuanced understanding of antithrombotic therapy in TBI [19,20].

Despite extensive research on the impact of VKA therapy versus DOACs on TBI outcomes, the results are contradictory. Some studies suggest a lower risk of spontaneous ICH with DOACs than VKA [21,22,23], while others report the contrary [24,25]. Over time, an increasing number of studies have reported similar risks of intracranial hemorrhage and mortality in patients using DOACs compared to those on VKA [12,13,14].

Some studies have shown that patients on antiplatelet therapy, such as Clopidogrel or dual antiplatelet therapy (aspirin with Clopidogrel), are more likely to experience traumatic ICH progression and require neurosurgical intervention, although the association with mortality is less clear [26,27]. A systematic review concluded that dual antiplatelet therapy increased the risk of ICH progression and the need for neurosurgery [28]. Conversely, a recent study indicated that patients using antiplatelet drugs prior to injury do not experience higher early mortality rates compared with patients who are not on any antiplatelet or anticoagulant medication, and that their use was not associated with an increased need for neurosurgical intervention and prolonged length of hospital stay [29].

Our study aims to delve into these complexities, particularly focusing on DOACs in the context of TBI in the emergency department, to provide clearer insights and to aid in the development of more effective treatment strategies. The primary aim is to evaluate the impact of DOACs on TBI outcomes, as well as to assess the incidence of intracranial hemorrhage (ICH) and the length of hospital stay for patients with TBI, and to compare these outcomes with those associated with other antithrombotic agents, such as VKA and antiplatelet drugs. A crucial aspect of the study is to analyze and contrast the risk of intracranial bleeding among different antithrombotic therapies, thereby determining which treatments may pose higher risks in the context of brain injuries. Furthermore, the study seeks to investigate the relationship between antithrombotic agents and the duration of hospitalization in TBI patients, an essential factor in patient management and healthcare resource planning.

## 2. Materials and Methods

Patients were retrospectively recruited from the Emergency Department of the Humanitas Clinical and Research Center in Rozzano, Milan, Italy, between 1 January 2021 and 28 December 2022. The study was approved by the ethical committee of Humanitas and performed in accordance with the principles of the Helsinki declaration (Protocol no. 369/20; approved on 22 April 2020). Informed consent was not required because of the registry nature of the study.

The inclusion criteria were a diagnosis of mild traumatic brain injury (TBI), defined by a Glasgow Coma Scale score ranging from 13 to 15, assessed 30 min after the injury occurred and involving one or more of the following indicators: a loss of consciousness lasting less than 30 min; post-traumatic amnesia not exceeding 24 h; a compromised mental state at the time of the accident, such as confusion or disorientation; and/or brief neurological deficits [30] in all age groups, both in patients who had and had not received prehospital antithrombotic agents.

At admission, the following data were collected for each patient as available: sex, age, and comorbidities, including a range of conditions such as cardiovascular disease, diabetes, cancer, chronic kidney disease, and others. Concurrent antithrombotic therapy included medications like aspirin (ASA), antiplatelet drugs (Clopidogrel, Ticlopidine, Plactadil, Ticagrelor), VKA (Warfarin, Acenocoumarol), DOACs (Edoxaban, Rivaroxaban, Apixaban, Dabigatran), and low-molecular-weight heparin (LMWH). Head CT scans were performed at 0, 6 h, 12 h, and 24 h from the time of arrival. Indication for follow-up CTs was based on individual clinical decisions in the case of neurological symptom onset during observation or in the event of bleeding detected by CT. Laboratory tests, including the international normalized ratio (INR), the blood concentration of the respective DOACs, creatinine and hemoglobin (Hb) levels, and the platelet count were collected.

The study’s primary outcome was the occurrence of ICH, and the secondary outcome was the length of hospital stay. Patient comorbidities were categorized into groups such as cardiovascular diseases (including conditions like atrial fibrillation and heart failure), malignancies (including various types of carcinomas), pulmonary diseases (like COPD and asthma), neurological disorders (including stroke and multiple sclerosis), and others.

### Statistical Analysis

The analysis was conducted using logistic regression models in R and STATA 13.1 software. Due to the lack of intracranial bleeding in patients using VKA and DOAC, these groups’ odds ratios (ORs) for their bleeding risk could not be calculated. Given the small sample size, Fisher’s exact test was employed to determine statistical significance. ORs were calculated by linear and logistic regression analysis to predict the length of hospital stay and to compare bleeding risks between ASA and other antithrombotic therapy groups.

## 3. Results

A total of 267 patients with mild TBI participated in the study. Table 1 displays the demographic and clinical characteristics of the study cohort. The mean age was 67.7 (±22.1) years, with a slight female predominance (53.9%). Out of the total population examined, 148 patients were not on antithrombotic agents, 43 were on aspirin only, 33 were on DOACs (6 of whom were also on aspirin), 5 were on LMWH (with 1 also on aspirin), 22 were on antiplatelets (5 of whom were also on aspirin), and 16 were on VKA. The most common indication for antithrombotic therapy was atrial fibrillation (Table 1).

The antithrombotic groups showed a significant difference in age with respect to the group not on antithrombotic therapy (*p* < 0.001). The average ages for patients not on antithrombotic agents and those on VKA, DOAC, antiplatelets, and LMWH were 58, 86, 83, 81, and 82 years, respectively. The most common comorbidities were cardiovascular diseases (56.6%), neurological conditions (18.7%), and diabetes (13.5%). The median INR for the entire cohort was 1.08, with VKA users having an average INR of 2.7, indicating effective therapeutic management. The median platelet count was 229,000 mmc, and hemoglobin was 13.1 g/dL, both within normal ranges. The median creatinine level was 0.89 mg/dL, with 32% of patients showing elevated levels (defined as creatinine > 1.00 mg/dL).

Out of the 267 patients admitted to the Emergency Room, 89.5% underwent a head CT scan during their ER stay, 65.2% at the time of admission, and 24.7% as a follow-up after 24 h (Table 2). Out of the 267 patients, 9 (3.4%) experienced intracranial bleeding, and only 1 patient required surgical intervention. Among those who experienced intracranial bleeding, the incidences of petechial hemorrhages, subdural hematomas, and epidural hematomas were 2.25%, 1.1%, and 1.1%, respectively. None of the patients on DOACs, LMWH, or VKA experienced intracranial bleeding, while 4 out of 65 on antiplatelets, and 5 out of 148 not on antithrombotic agents did (Table 2).

Due to the absence of bleeding cases in the VKA and DOAC groups, statistical analysis for the intracranial bleeding OR was not feasible. However, the odds ratio (OR) for predicting bleeding in patients on ASA was higher at 1.98 (95% CI: 0.48–8.18), while it was notably lower at 1.03 (95% CI: 0.8–1.33) in the anticoagulant/antiplatelet group (Table 3). These results remained consistent even after adjusting for sex, age, and comorbidities, with a wide 95% CI in both cases (Table 3).

Table 4 presents the average length of hospital stay (LOS) for patients with traumatic brain injury (TBI), differentiated by the type of antithrombotic agent used. Patients not on any antithrombotic therapy (None) had the shortest average LOS at 0.46 days (±2.73), indicating relatively brief hospitalizations. Those on antiplatelet therapy had a slightly longer average LOS of 0.73 days (±0.83). Patients treated with DOACs showed a further increase in the LOS, averaging 0.97 days (±0.68). Those on vitamin K antagonists (VKA) had the longest average LOS at 1.19 days (±0.75). This data suggests a trend where the LOS tends to increase with the use of more intensive antithrombotic therapies, with VKA-treated patients staying the longest in the hospital following a TBI.

Table 5 presents the ORs for the length of hospital stay following TBI in relation to different antithrombotic agents, both before and after adjusting for sex and age. In the unadjusted analysis, patients on VKA had the highest OR for a longer hospital stay at 2.1 (95% CI: 0.7–6.1), though this was not statistically significant (*p* = 0.185). Similar trends were observed for patients on DOAC and LMWH, with ORs of 1.66 and 1.71, respectively. Antiplatelet therapy had an OR of 1.3, and ASA monotherapy had the lowest OR at 1.15, indicating the least likelihood of a prolonged hospital stay among the groups studied.

After adjusting for sex and age, the ORs remained largely unchanged, indicating that these demographic factors did not significantly impact the length of stay associated with different antithrombotic therapies.

## 4. Discussion

In the present study, the impact of various antithrombotic agents on patients with mild TBI was investigated. Four cases of bleeding among patients on antiplatelet therapy, two on aspirin monotherapy, one on Clopidogrel, and another on a combination of aspirin and Clopidogrel were found, while five other bleeding cases were observed in the group not on any therapy. This aligns with some existing literature, which suggests an increased risk of bleeding with aspirin as a monotherapy [31] and with the combination of aspirin and Clopidogrel [32]. Another study reported similar findings, with aspirin showing the highest risk for ICH compared with VKA, DOACs, and antiplatelets [12]. It was hypothesized that this might be due to patients on aspirin being frailer or the possibility of aspirin itself carrying the highest risk of ICH [12]. Interestingly, none of our patients on VKA or DOACs experienced bleeding following TBI. This contrasts with other studies where VKA-treated patients exhibited a higher prevalence of severe head injury and mortality with respect to controls and other antithrombotic therapies [33,34]. The debate over the comparative risks of VKA and DOACs remains contentious, with some research showing conflicting results [21,23,25], while other studies find no significant difference compared to VKA [12,13,14,35]. With regards to the increased bleeding found in our group not on anticoagulation or antiplatelet therapy, other studies also found that the incidence of traumatic intracranial hemorrhage was not increased in those taking these medications compared to those not taking them [36].

It is important to note that while aspirin showed a higher likelihood of predicting intracranial bleeding compared with the anticoagulant/antiplatelet group, these results were not statistically significant, aligning with findings reported by another study, primarily due to the wide 95% confidence interval (CI) [37]. This suggests that there may not be an increased risk of bleeding in the aspirin group relative to the antiplatelet/anticoagulant group, a conclusion that could be attributed to our study’s small sample size of patients who experienced bleeding from a mild TBI.

Interestingly, compared with other studies, we observed a lower overall intracranial bleeding rate, which might be attributed to different definitions of mild TBI and local protocols for obtaining head CT scans [38,39]. European guidelines advocate for a mandatory CT scan and a period of observation for all patients with head injuries on anticoagulation therapy, irrespective of their clinical symptoms [40]. In contrast, in the United States, the most commonly applied clinical criteria for deciding on the need for a CT scan, such as the New Orleans Criteria and the Canadian CT Head Rules, do not specifically address patients on anticoagulant therapy [41,42]. Furthermore, while major guidelines recommend considering CT scans in cases of coagulopathy, they also acknowledge the lack of sufficient data to support the safe discharge of mild TBI patients on oral anticoagulation who have negative initial CT scans [43]. This uncertainty is compounded by the limited information regarding the incidence and prevalence of delayed traumatic intracranial hemorrhage, leading to ongoing debate over the most appropriate management approach for these patients.

Regarding the length of hospital stay, literature evidence reports that this would be related to multiple factors, especially during the COVID-19 pandemic [44,45,46]. Several factors could contribute to the lack of a significant association observed between antithrombotic therapy and the length of hospital stay. Possible reasons might include the variability in patient demographics and clinical profiles, such as the presence of comorbidities, as well as the heterogeneity in antithrombotic therapy protocols.

However, the overall trend of prolonged duration for patients on VKA and DOACs aligns with Italian guidelines that mandate a 24 h observation window and a second CT scan before discharge for all anticoagulated patients [47,48]. This guideline is based on the known incidence of delayed ICH, which can occur even after an initially negative CT scan [47,48]; reported incidences of this range from 0% to 6%.

The existing literature offers mixed insights on this matter, with some studies corroborating our findings on extended hospitalization for VKA users, while others report no significant differences between VKA and DOACs [15,25]. These discrepancies could be due to differences in study populations, the severity of TBI, or healthcare practices in various regions.

Although the bleeding risk from antithrombotic therapy affects more moderate and severe TBIs, the aim of the present study focuses on patients with mild TBI, which is driven by a specific research gap we identified in the current literature. Patients from the mild TBI group, while less likely to suffer from major intracranial bleeding compared with those with moderate or severe TBIs, still represent a significant clinical challenge due to the subtleties involved in their management and the potential consequences of antithrombotic therapy [2]. The clinical implications of our study, alongside the broader literature, emphasize the need for individualized patient assessment when prescribing antithrombotic therapy, especially for those at risk of TBI. The necessity for further research is clear, particularly in larger-scale studies, for more definitive conclusions on the risks and benefits of various antithrombotic therapies in TBI patients.

Overall, this study underscores the complexity of managing TBI in patients on antithrombotic therapy and emphasizes the need for individualized patient assessment. The ongoing debate and the necessity for more extensive research, particularly on newer anticoagulants like DOACs, highlight the evolving nature of clinical practices in this area. Our findings add valuable insights to the discussion about the safety and efficacy of antithrombotic agents in TBI patients, pointing toward the need for continuous evaluation and adaptation of clinical practices as new evidence emerges.

### Strengths and Limitations

The study’s strength lies in its inclusive approach, enrolling all patients who entered the ER within our specified timeframe, thus reducing selection bias. Recording patient comorbidities, including kidney function and relevant lab values (platelets, INR, hemoglobin), provided valuable insights into the bleeding risks of TBI patients upon arrival. However, being a retrospective study, it offers a lower level of evidence compared with prospective studies and is susceptible to various biases. Data extraction from medical records raises concerns about the accuracy of entries and the timing of the data input. In addition, the lack of detailed information about the specific types of trauma experienced by our patients with mild TBI prevents a more in-depth analysis of how different types of trauma may influence the outcomes of patients on antithrombotic therapy, potentially affecting the generalizability of our findings. Another significant limitation was the small sample size, especially as none of the patients in the DOAC and VKA groups experienced intracranial bleeding, preventing the calculation of an OR for these groups and affecting the attainment of statistically significant results. These preliminary findings suggest a need for further studies, possibly through multicenter collaborations, to verify the results and establish statistical significance.

## 5. Conclusions

This study aimed to evaluate the outcomes of TBI patients who were on antithrombotic therapy, focusing on two key aspects: their length of hospital stay and the incidence of ICH. Our findings indicate that TBI patients on pre-injury anticoagulants/antiplatelets generally had longer hospital stays compared with those taking only aspirin. Notably, VKA emerged as the strongest predictor for an extended hospital stay. DOACs also showed longer stays compared with LMWH, while only antiplatelets were associated with a shorter predicted length of stay. However, due to the small sizes of our subanalysis cohorts, these results were not statistically significant. This lack of significance, coupled with conflicting findings in existing literature, underscores the need for further research to clarify these relationships. Regarding the predictors of ICH in TBI patients, our study found that individuals taking only aspirin were twice as likely to experience bleeding compared with those on anticoagulants/antiplatelets. While similar results have been reported in other studies, we believe that our findings might be attributable to the small sample size, as indicated by the wide 95% confidence interval. Therefore, to achieve statistically significant results, further research with a larger cohort of patients is necessary.

Across the board, this study contributes to the understanding of how antithrombotic agents affect TBI patient outcomes, particularly in terms of hospital-stay length and bleeding risk. It adds valuable insights to the ongoing discussion about the safety and efficacy of various antithrombotic agents in TBI patients. It highlights the complex balance between the therapeutic benefits of these drugs and their potential risks, particularly in vulnerable populations like those with TBI. The discussion also points toward the continuous need to evaluate and adapt clinical practices as new evidence and treatments become available.

## Figures and Tables

**Table 1:** Demographic and clinical characteristics of the study cohort.

	Total (*n* = 267)
Age (years), median (IQR)	76 (50–85)
Female, *n* (%)	144 (53.9)
*Comorbidities*	
Cardiovascular, *n* (%)	151 (56.6)
Diabetes, *n* (%)	36 (13.5)
Cancer, *n* (%)	17 (6.4)
Chronic kidney disease, *n* (%)	9 (3.4)
Lung disease, *n* (%)	15 (5.6)
Neurological disease, *n* (%)	50 (18.7)
Endocrinologic disease, *n* (%)	27 (10.1)
Gastrointestinal and liver disease, *n* (%)	21 (7.9)
Psychiatric disease, *n* (%)	13 (4.9)
Vascular disease, *n* (%)	5 (1.9)
Opthalmologic disease, *n* (%)	10 (3.7)
Rheumatic disease, *n* (%)	7 (2.6)
*Anticoagulants and antiplatelets drugs*	
**Antiplatelet, *n* (%)**	65 (24.3)
ASA, *n* (%)	43 (16.1)
Clopidogrel, *n* (%)	10 (5.6)
Clopidogrel and ASA, *n* (%)	5 (1.9)
Ticlopidine, *n* (%)	5 (1.9)
Plactadil, *n* (%)	1 (0.4)
Ticagrelor, *n* (%)	1 (0.4)
**DOACs, *n* (%)**	33 (12.4)
Rivaroxaban, *n* (%)	4 (1.5)
Rivaroxaban and ASA, *n* (%)	1 (0.4)
Edoxaban, *n* (%)	10 (3.7)
Apixaban, *n* (%)	9 (3.4)
Apixaban and ASA, *n* (%)	3 (1.1)
Dabigatran, *n* (%)	4 (1.5)
Dabigatran and ASA, *n* (%)	2 (0.7)
**VKAs, *n* (%)**	16 (6.0)
Warfarin, *n* (%)	15 (5.6)
Acenocoumarol, *n* (%)	1 (0.4)
**LMWH, *n* (%)**	5 (1.9)
Enoxaparin, *n* (%)	4 (0.4)
Enoxaparin and ASA, *n* (%)	1 (0.4)
**None, *n* (%)**	148 (55.4)

**Table 2:** Outcomes, imaging, and bleeding distribution based on the antithrombotic agent.

Category	Total (*n* = 267)
**Death, *n* (%)**	0
**Surgical intervention, *n* (%)**	1 (0.4)
**Bleeding, *n* (%)**	9 (3.4)
Subdural hematoma, *n* (%)	3 (1.1)
Epidural hematoma, *n* (%)	0
Subarachnoid hemorrhage, *n* (%)	3 (1.1)
Intraparenchymal hemorrhage, *n* (%)	0
Petechial hemorrhage, *n* (%)	6 (2.25)
**Bleeding Distribution by Antithrombotic Agent**	
Antiplatelet, *n* (%)	4/65 (6.2)
ASA alone, *n* (%)	2/43 (4.7)
Clopidogrel alone, *n* (%)	1/10 (10)
Clopidogrel and ASA, *n* (%)	1/5 (20)
DOAC, *n* (%)	0/33 (0)
LMWH, *n* (%)	0/5 (0)
None, *n* (%)	5/148 (3.4)
VKA, *n* (%)	0/16 (0)
**Length of stay (days), mean (SD)**	0.6 (2)
**Brain CT (any time), *n* (%)**	239 (89.5)
Brain CT at admission, *n* (%)	174 (65.2)
Brain CT follow-up at 6 h, *n* (%)	42 (18.5)
Brain CT follow-up at 12 h, *n* (%)	34 (12.7)
Brain CT follow-up at 24 h, *n* (%)	66 (24.7)

**Table 3:** Predictors of bleeding in ASA vs. anticoagulants/antiplatelets groups.

	OR, 95% CI (*p* Value)
*unadjusted*	
ASA	1.98, 0.48–8.18 (0.34)
Anticoagulants/antiplatelets	1.03, 0.8–1.33 (0.8)
*adjusted for sex and age*	
ASA	1.99, 0.41–9.71 (0.39)
Anticoagulants/antiplatelets	1.00, 0.75–1.34 (0.98)

**Table 4:** Average length of stay in days at the hospital based on antithrombotic agents.

Antithrombotic Agent	Average LOS (Days in Mean ± SD)
None	0.46 ± 2.73
Antiplatelet	0.73 ± 0.83
DOAC	0.97 ± 0.68
LMWH	1
VKA	1.19 ± 0.75

**Table 5:** Length of stay for different antithrombotic agents.

	OR (95% CI)	*p* Value
*unadjusted*		
ASA monotherapy	1.15 (0.6–2.1)	0.630
Antiplatelet	1.3 (0.5–3.1)	0.574
DOAC	1.66 (0.8–3.6)	0.200
LMWH	1.71 (0.26–11.1)	0.572
VKA	2.1 (0.7–6.1)	0.185
*adjusted for sex and age*		
ASA monotherapy	0.6 (0.3–1.2)	0.150
Antiplatelet	1.3 (0.5–3.5)	0.588
DOAC	1.6 (0.7–3.8)	0.248
LMWH	1.6 (0.2–11)	0.600
VKA	2.1 (0.7–6.7)	0.193

## Data Availability

The data that support the findings of this study are available on request from the corresponding author.
